# Finding your lane: experiences and beyond for adults learning to swim

**DOI:** 10.1186/s12889-023-17320-0

**Published:** 2023-12-07

**Authors:** Shawn Wilson, Alison Moira Miller, Destiny Casson, William D. Ramos

**Affiliations:** 1 School of Medicine, Indiana University, Indianapolis, IN USA; 2 Health and Wellness Design, School of Public Health, Indiana University, Bloomington, IN USA

**Keywords:** Swimming, Adults, Safety, Motivations, Experience, Water, Aquatics, Instruction, Swim lessons

## Abstract

The purpose of this study was to examine experiences and impacts from participating in an adult swim instruction program. We conducted 20 semi-structured interviews with adults aged 18 + who had participated in an adult learn to swim program. Participants were interviewed through a virtual platform using a semi-structured protocol. Data were coded individually by each member of the research team for emerging thematic outcomes with final consensus among all those involved in the analysis. Participants expressed adult learn to swim programs had an effect in three areas: (a) life affordance, (b) emotional affect, and (c) interpersonal relationships. Public health experts and aquatics leaders should consider targeting programs aimed at teaching adults to learn how to swim, especially to marginalized individuals and those who did not grow up in the United States. The authors highlight how these programs can create life affordances well beyond lap swimming and even water safety.

**Supplementary Information:**

The online version contains supplementary material available at 10.1186/s12889-023-17320-0.

## Background

Aquatic activities are popular leisure pursuits across the globe, from swimming pools, oceans, lakes, and rivers, there is a water-based activity for all throughout the lifespan. Aquatic pursuits can also promote personal and population health through physical activity, social connections, and sense of wellbeing [1]. Although aquatic activities offer many potential benefits, individuals who do not know how to swim are at risk for drowning [2, 3]. Annually in the United States there are an estimated 3,960 fatal unintentional drownings, with an average of 11 drowning deaths per day [4]. Drowning is the third leading cause of unintentional injury death worldwide, accounting for 7% of all injury-related deaths [5]. Approximately 37% of the U.S. adult population reports limited swimming ability [6], and about one-third of U.S. adults cannot swim the length of a pool [4]. Additionally, a 2015 analysis found that one-third of swimming pool drowning deaths were middle-aged (45–64 years) and older-aged (65 + years) adults [7]. These public health trends related to limited swimming ability can have lasting impacts on health and wellbeing. From 2015 to 2020 there was a 41.8% increase in drowning research publications [8]. Past research efforts examine risk factors related to drowning concerning demographic data, swim lessons impact on drowning prevention, as well as predictors of swimming ability; however, little research has been done regarding how learning to swim can impact the lives of adults who did not previously have this valuable life skill. There are also data identifying inequities in drowning prevention resources related to socioeconomic and racial factors [9]. Through this study we can hopefully elucidate on an individual level why adults are motivated to learn to swim later in life.

### Barriers and predictors of swimming ability

There are various reasons why adults do not learn to swim as children or identify with having low swimming competence [10]. Variables positively associated with swimming ability include the ability of parent(s) to swim, child/adolescent age, a best friend who enjoys swimming, water-safety knowledge, pool being open all year, and encouragement to swim from parent(s). Variables negatively associated with swimming ability include fear of drowning and lack of access to pools in community due to legal and de facto segregation [11]. These variables begin in childhood, impacting and predicting if an adult knows how to swim and feels competent in that ability. By looking at these variables, we can try to focus on the strengths of positive association and targeted interventions while also examining the root issues of negative association. A 2007 study of 18–35-year-olds found that these individuals overestimated their swimming abilities [12]. Furthermore, this finding persists through the lifespan. A study of adults aged 65 + found that those who believe their swimming abilities are stronger than they are may underestimate their drowning risk and may also lack a clear understanding of drowning probability [13] (Abercromby et al., 2022). Inequity also factors into swimming ability.

Access to drowning prevention resources is inequitably distributed within communities in the United States. For example, demographic factors are significant predictors of swimming ability. In a 2009 study, African American respondents reported a 57.5% “at risk” (unable to swim or uncomfortable in the deep end of the pool) swimming ability. Similarly, Hispanic/Latino children confirmed a 56.2% “at risk” level as compared with 30.9% for White subjects [14]. Furthermore, the drowning rate for American Indians/Alaska Natives is two times higher than the rates for White people [15]. African Americans and other ethnic minorities were historically barred from access to pools because of racist policies. Although these policies no longer exist, equitable access to aquatics as a leisure pursuit and learning the skill of swimming for the purposes of drowning prevention are continually impacted [16]. Being male, African American, and low- income are also predictive of drowning risk [9]. In a 2020 study of 4,355 drowning related hospital admissions, 68.3% were male [17]. In addition, a 2020 study found that males aged 65–74 years were at increased risk of drowning in comparison with other adult characteristics or age ranges [18]. Beyond systemic barriers, it is also important to understand personal barriers to swimming.

If an adult has a negative prior aquatic experience, they are more likely to have a fear of water and a lower achievement and progress during swim lessons [19]. A 2021 study of adult women found that fear of water was a common theme attributed to a previous “drowning” experience and not knowing how to swim [19]. According to a 2020 study, 4% of those surveyed had experienced a negative prior aquatic experience, and of those, 19% were from swim lessons [20]. Because 19% of these originated from swimming lessons, this could be addressed by training adult swim instruction program instructors how to address fear and more appropriately in the water in order to help individuals to become comfortable with swimming.

### Adult learning theory

Knowles proposed a theory of adult learning, called *andragogy*, meaning “the art and science of helping adults learn” [21]. Knowles’ theory was based on the characteristics that distinguish the mature adult from the pre-adult learner, including: (a) self-directedness, (b) accumulated reservoir of experience that becomes a resource for learning, (c) readiness to learn and, (d) application of knowledge, (e) internal motivation to learn, and (f) the need to know why something should be learned. Knowles’ theory is an important underpinning to our study because participants often came to the adult learn to swim program with a readiness to learn, internal motivation to learn, and knowing why it was important to learn to swim. Based on this theory, when it comes to learning to swim, an individual must overcome any negative past experiences or beliefs; believe they can accomplish something difficult; have a support network; and want to accomplish learning to swim for intrinsic reasons. Formosa revealed that older adults’ participation in learning activities has a positive impact on the participants’ levels of active ageing. Moreover, learning a new skill can help keep adults sharp both mentally and physically and could ultimately add a lifetime of wellbeing through a new leisure and physical activity pursuit [22, 23]. Andersen identified attributes in common between adults who learn a new skill later in life: aspiration, self-awareness, curiosity, and vulnerability. “They truly want to understand and master new skills; they see themselves very clearly; they constantly think of and ask good questions; and they tolerate their own mistakes as they move up the learning curve” [24]. 

### Breaking the cycle of drowning

We must understand the barriers that prevent individuals from learning to swim. Negative prior aquatic experiences as children is one important factor to be considered as they can affect one’s interest in learning to swim and perceived skill level. When adults have a negative aquatic experience there is an interplay between four psychological constructs: (a) past experiences, (b) difficulty or challenge associated with the outcome, (c) reinforcement and personal interactions with significant others, and (d) intrinsic motivation [25]. Adult swim instruction programs are one way to overcome negative aquatic experiences as they provide a psychologically and physically safe space for adults to become comfortable with water and overcome fears related to the water and swimming.

It is also important to acknowledge that not all hesitancy to swim is individual or related to negative prior aquatic experiences as children. In the United States, Jim Crow laws and racist practices created segregated pools leaving Black/African American children and families without access to most public pools for generations [26]. During the early-to-mid twentieth century, swimming pools and beaches were among the most segregated public spaces in the country [26, 27]. White residents advocated for segregation of public pools by spreading false rumors that African American swimmers would spread diseases to white swimmers. It was not until 1964 that the Civil Rights Act desegregated public swimming pools and parks. Although the law called for integration of swimming pools, some municipalities created clubs with membership fees to prevent African American patrons from entering. Others simply closed the city pools and filled them with concrete. During the 1960s and 1970s, many White families left cities in favor of suburbs. The rise of these affluent suburbs saw a dramatic increase in the number of gated communities, homeowners’ associations, and informally segregated private pools. As private swimming pools became more popular, cities began to decrease their funding to public recreational facilities and school pools, further preventing African American patrons from access to pools. Given the legal segregation and de facto exclusion from public swimming pools, adult swim instruction programs have the potential to break down cultural barriers related to swimming. In a 2014 study, African American adult participants repeatedly stated their fear of water was “passed down” and there were numerous examples of how fear of swimming were passed from generation to generation, including forbidding children to attend school-sponsored trips to waterparks [16]. A 2014 study found that rather than viewing swim lessons as a means of protecting their children by making them safe in and around water, African American parents who had a fear of water themselves hoped to keep their children out of the water altogether believing this to be a mode of protection instead [28]. Some parents in this study shared attitudes of determination and willingness to conquer fears or overcome obstacles that have kept them, and could have prohibited their children, from learning to swim. The parents who tried to learn how to swim as adults mentioned that they wanted to inspire their children [28]. The authors of this study were interested to see if adult swim instruction programs could help adults to overcome personal and systemic barriers as well as how adult swim instruction programs provides other life affordances.

The purpose of this study was to examine experiences and impacts from participating in an adult swim instruction program. The United States Masters Swimming Adult Learn to Swim (ALTS) program is designed for community members who are interested in learning to swim. Instructors are generally member volunteers with a local master’s program. Adult swim instruction programs can help fill the gap of individuals who never had the opportunity to learn to swim as a child and would like to learn now as an adult and although not a requirement, many of the participants in the program are members of marginalized communities. The authors sought to understand the experiences and motivations of adults that learn to swim later in life. 

## Methods

### Approach

Our qualitative study employed semi-structured interviews as a basis for data collection. Qualitative interviews are a popular research method in social science research that aims to uncover participants’ experiences, attitudes, and beliefs about a particular phenomenon [29, 30]. The study consisted of 20 semi-structured interviews conducted virtually and recorded for transcription and analysis. Inclusion criteria for the study involved adults, 18 years of age or older, who had been pre-identified as having participated in at least one session of an adult swim instruction program.

The research team was comprised of four individuals with a background in social science research and qualitative research. Two faculty members, one from medicine and one from public health led the design and implementation of the study. Together, they have more than 30 years of experience as qualitative researchers. Additionally, two doctoral students participated in the interviews and analysis of the data. Our team was also diverse in terms of gender, age, and race/ethnicity.A local masters swimming program in the Midwest, operated by a member organization of United States Masters Swim (USMS), was used as the primary source for potential study participants. Contact information for pre-identifying program participants was obtained with consent of the study site organization with potential subjects receiving information about the study through email correspondence. Once participants expressed interest, a member of the research team coordinated the interviews.

Semi-structured interviews were conducted by members of the research team and transcribed using Zoom. Interviews were held between February and June of 2022 and scheduled for 45-min. Permission was obtained from study subjects to record interviews. A recent study highlighted the benefits of using videoconferencing technology, which can overcome barriers to participation and reach a wider range of participants [29]. Given that our participants lived all over the United States and work schedules varied, virtual interviews allowed for the broadest participation. The study was conducted as an exempt protocol, approved by the Institutional Review Board of Indiana University (13576, December 14, 2021).

### Research setting

Participants for this sample were recruited from a USMS Adult Learn-to-Swim program in the Midwest region of the US. Subjects included registered program participants between 2014–2021. Subjects were recruited from an existing registration database, contacted via email and invited to respond about participation. Participants were only contacted to participate after completing the swimming program. Based on the nature of the program described above, swim instructors do not have due influence over the participants.

### Data collection and sample

Participants who completed at least one session were recruited for interviews to understand their experiences, motivations, and outcomes of participating in an adult swim instruction program. Participants were asked to voluntarily share their age, gender and race/ethnicity. Among the participants, 11 self-identified as female and 9 as male. Furthermore, seven participants self-identified as Black/African American, five as White non-Hispanic, two as Hispanic/Latino, three as Indian, two as Asian, and one as Native American/White.

The research team partnered with USMS swim program leaders who provided the roster and contact information for individuals who had previously participated in the program. The research team contacted individuals via e-mail and invited them to patriciate in a virtual interview. Before scheduling, participants were provided information about the study and were informed about a gift card incentive for participation.

### Data interpretation

Inductive thematic analysis was used to construct a variety of themes from the data. The interview transcripts were read and a coding taxonomy was developed. Following two distinct readings of the transcript data, codes were identified and grouped, leading the creation of themes [29]. Raw data were analyzed through a process sometimes referred to as “open coding,” wherein conceptual categories were identified and tentatively named according to the observed phenomena [31]. Finally, subthemes were mapped in accordance with our research aim. When necessary, the audio recordings were used to provide context to what was said. All authors read and coded every interview transcript and identified their own initial themes. The authors then came together on several occasions to discuss initial codes. The authors developed an agreed set of codes and then went back to the data to confirm that data were coded consistently among raters.

## Results

### Themes

Themes were grouped into three categories: (1) life affordance, (2) emotional affect, and (3) interpersonal relationships. Representative quotes are presented for each theme.

### Life affordance

For the purpose of this research study life affordance is defined as what the environment offers the individual, in this case the ability to swim can expand life experiences and opportunities that would otherwise be inaccessible. This section explores the pursuit of life affordances as a motivating factor for learning to swim. Learning to swim provided participants not only with swimming, but also a wide variety of water-based activities. For some, participating in the program went beyond just the water and reminded them of the importance of staying active and learning new things later in life. Many acknowledged their motivation to learn to swim was fueled by a desire to feel confident pursuing water-based recreation and leisure activities. For example, a participant, an Indian male in his 20s, described his motivation and life affordance this way, that they “want to do some enjoyable activities like scuba while I travel, that would be something I would want to pursue to enjoy my life.” Another participant, a White male in his 20s, explained how the program helped him overcome his fear, “the program helped me become more adventurous for sure, because I went from never being able to swim to doing shark cage diving at night and diving with manta rays in Hawaii.” Having swimming competence provided these participants with enjoyable life experiences they would not have otherwise been able to partake in. These life affordances can broaden horizons and open possibilities that were previously unavailable.

Life affordances and motivations related to water-based physical activity opportunities were also referenced by participants. One participant, a Black/African American female in their 40s explained, “You know, with exercise I’m able to get exercise, and I could see myself doing like water aerobics at my gym and things that I maybe would not have been able to do…and I’m super proud of myself and that would not have happened if I hadn’t taken that course.” Another participant, a White female in their 30s said, “So I wanted an activity an exercise activity as well, so I did the lessons and then that winter I got a membership at the gym for the indoor pool, and I did swim all that winter.” These examples illustrate the impact feeling comfortable in the water can have on recreational and physical activity choices. Having the ability to participate in water-based physical activity, in addition to other life affordance opportunities, has the potential to positively impact additional health outcomes as well as behaviors, creating a ripple effect on personal wellbeing.

### Emotional affect

Swimming or the idea of swimming and aquatics-based activities elicited many different emotions for participants. Participants reported their experience learning to swim as frustrating, nervous, anxious, and intimidating. Participants indicated that not knowing how to swim was often deeply rooted in visceral fear of water and drowning. As a result of the program, participants shared feelings of excitement, accomplishment, and confidence.

Most participants shared emotions and beliefs about water that went back to childhood. Many of these participants shared some of the emotions that caused them to avoid swimming and water, often long into adulthood. For example, a participant, an Indian male participant in his 30s shared, “I think for me, as with a lot of other participants, it was a fear of swimming.” He went on to share his experience swimming as a child by saying, “I took some classes in high school, but I still had this pretty drastic fear of the water. So, that is what it was for me, I think, it was based on this inability that I felt I had, and I feel like the program helped.”

For many, the program was transformational and altered for the better their perceptions A participant, a Black/African American male in his 20s explained, “I remember very vividly the instructor had taken us over to the deep end. He wanted us to jump into the water. I was like, I don’t know if I want to do this.” He went on to share that after going through a couple of classes he jumped into the deep end of the pool and said, “I remember jumping and was like ‘it’s not that bad.’ I won’t forget that. It was unreal. I have skydived before and all this other stuff. I don’t know what it was about it, but I did it. I think that helped me progress to another level.” For another participant, a White male in his 60s, the program helped him to gain confidence. He said, “if there is something you think you want to do then no matter how hard it is, you should go ahead and do it. For me there was a lot of confidence building (from completing the swim program).” This program not only helped participants overcome barriers, but it also built confidence for themselves and had a ripple effect on others in their lives.

### Interpersonal relationships

Participants’ interpersonal relationships served as a prominent reason for deciding to complete an adult swim instruction program. For many participants, having parents or family members who could not swim played a large role in them not learning how to swim as children. Participants indicated that swimming was rarely discussed in their families and communities. For example, a White and Native American male in their 60s shared, “as a kid, I had a father who was terrified of water. His mother was terrified of water too. My dad said that his mother was afraid even to let him or his brother take a bath because she was that terrified of water.” For many participants, they grew up in families where their parents, grandparents, and other adults in their life did not swim. As a result, they were not exposed to water-based activities or did not take swimming lessons.

Interestingly, it was also interpersonal relationships that caused many adults to decide they wanted to learn how to swim. For example, one participant shared that she was now a grandmother and she wanted to feel comfortable taking her grandchildren to the pool to swim. Similarly, several participants were parents and they wanted to make sure their children could swim, so they could fully participate in social activities with their friends. For example, a Black female in her 40s, and a mother of four, shared how the experience influenced her adult cousin to decide to swim, “she doesn’t know how to swim very well either. On vacation I told her about my experience with my swim class and now she is into it (where she lives).” The experience also helped this participant connect with her adult children. “There was kind of trickle down and also my kids—I have four adult kids—they all know how to swim, and I was like the only one in the family that didn’t know how to swim. They were really proud of me for learning to swim and now we are talking about planning a vacation to beach as a family and enjoying the water together.” As she reflected on the past year she said, “You know I have done a lot of transformation this year so I am like a new mom.”

## Discussion

The experiences and impacts of an adult swim instruction program were examined by conducting 20 semi-structured interviews with adults. The participants expressed that the program had a positive effect in three areas: life affordance, emotional affect, and interpersonal relationships. The intentional design of this programs acknowledges the unique needs highlighted in adult learning (e.g. internal motivation to learn, reservoir of experience that becomes a resource for learning) [21].

### Life affordance

The results of this work may inform the importance of water safety programs directed towards teaching adults to swim. Regardless of whether they continued to swim as a form of exercise, the process of learning how to swim and overcoming the fear of water opened doors not only for participants but also for others in their lives [9]. Furthermore, as individuals age, they may realize there is an activity they would like to gain competency in that would grant them benefits such as wellbeing, life affordances, and quality of life [32]. Public health experts and aquatics leaders should consider targeting adults for swim education programs, especially minoritized marginalized individuals and those who did not grow up in the United States – all of which can have a significant impact on reducing health disparities in drowning.

### Emotional affect

Many participants entered the program with previous experiences that influenced their emotional response to aquatic activity. Adult learning theory provides a framework for understanding internal motivation and readiness to learn [21]. These experiences were typically negative, creating a barrier to participation [19]. Once this barrier was overcome and during participation in the program, participants expressed a shift in their feelings towards water. Positive experiences were created by the program instructors that, in turn, initiated positive emotional responses. Two vital factors in this process includes trusting relationships and empowering individuals in the aquatic learning environment [33]. Future research should focus on creating a unified instructional approach to addressing fear and other negative emotions associated with aquatic activities.

### Interpersonal relationships

Although the goal of the Adult Learn to Swim program is to teach adults the skills to swim, the benefits of this program seem to span beyond the lane lines of the pool [24]. In fact, the ripple effect of interpersonal relationships was an important secondary outcome of this program. For example, many participants went on to encourage their children, siblings, and other family members to take swimming lessons after they did so themselves, which can help teach future generations to swim and ultimately disrupt the cycle of drowning. Existing research points to many adults in the United States, especially racially minoritized and immigrants, who do not know how to swim. The program was largely able to serve minoritized groups given the proximal location of the pools in which the program was offered—thus, addressing some of the historical barriers mentioned in the literature [14, 16, 33]. It is paramount that adult swim instruction programs continue to be offered and accessible.

### Limitations

A limitation of the study was the number of participants. Although this study was held over a 5-year period, consistent contact between participants and program leaders at the conclusion of the program was a challenge. Another limitation concerned our research team itself, which did not include anyone who was born out of the United States. Additionally, no one from Black/African American or Indigenous/Native American populations participated in the data collection or analysis, so our ability to understand the lived experiences of participants from these backgrounds was limited. Future studies could explore the experiences of the volunteers who teach this program, as well as replicate this study at various locations in the United States.

## Conclusion

This study provides valuable insights for program developers and public health officials on the potential benefits of adult swim instruction programs beyond just swimming skills and safety. The authors emphasize that these programs can create life affordances beyond lap swimming and water safety. Further research can expand on the study’s findings and explore other potential benefits of adult swim instruction programs.

### Supplementary Information

**Additional file 1.**

## Data Availability

Data is not publicly available. For further information about the relevant interview transcripts notes please contact the corresponding author.
